# Systematic gene therapy derived from an investigative study of AAV2/8 vector gene therapy for Fabry disease

**DOI:** 10.1186/s13023-023-02894-0

**Published:** 2023-09-05

**Authors:** Mulan Deng, Hongyu Zhou, Shaomei He, Haoheng Qiu, Yanping Wang, April Yuanyi Zhao, Yunping Mu, Fanghong Li, Allan Zijian Zhao

**Affiliations:** The School of Biomedical and Pharmaceutical Sciences, Guangdong University of Technology, Guangzhou, 510006 Guangdong Province People’s Republic of China

**Keywords:** Fabry disease (FD), Adeno-associated viral 2/8 (AAV2/8), α-galactosidase A (α-Gal A), Globotriaosylceramide (Gb3), Globotriaosylsphingosine (Lyso-Gb3), Lysosomal storage diseases (LSDs)

## Abstract

**Background:**

Fabry disease (FD) is a progressive multisystemic disease characterized by a lysosomal enzyme deficiency. A lack of α-galactosidase A (α-Gal A) activity results in the progressive systemic accumulation of its substrates, including globotriaosylceramide (Gb3) and globotriaosylsphingosine (Lyso-Gb3), which results in renal, cardiac, and/or cerebrovascular disease and early death. Enzyme replacement therapy (ERT) is the current standard of care for FD; however, it has important limitations, including a low half-life, limited distribution, and requirement of lifelong biweekly infusions of recombinant enzymes.

**Methods:**

Herein, we evaluated a gene therapy approach using an episomal adeno-associated viral 2/8 (AAV2/8) vector that encodes the human *GLA* cDNA driven by a liver-specific expression cassette in a mouse model of FD that lacks α-Gal A activity and progressively accumulates Gb3 and Lyso-Gb3 in plasma and tissues.

**Results:**

A pharmacology and toxicology study showed that administration of AAV2/8-hGLA vectors (AAV2/8-hGLA) in FD mice without immunosuppression resulted in significantly increased plasma and tissue α-Gal A activity and substantially normalized Gb3 and Lyso-Gb3 content.

**Conclusions:**

Moreover, the plasma enzymatic activity of α-Gal A continued to be stably expressed for up to 38 weeks and sometimes even longer, indicating that AAV2/8-hGLA is effective in treating FD mice, and that α-Gal A is continuously and highly expressed in the liver, secreted into plasma, and absorbed by various tissues. These findings provide a basis for the clinical development of AAV2/8-hGLA.

**Supplementary Information:**

The online version contains supplementary material available at 10.1186/s13023-023-02894-0.

## Background

Fabry disease (FD, OMIM: 301500) is an X-linked inherited lysosomal storage disease (LSD) caused by mutations in the *GLA* gene that encodes the exogalactosyl hydrolase α-galactosidase A (α-Gal A; GenBank: NP_000160.1) and results in insufficient α-Gal A activity [1]. α-Gal A is synthesized as a 50 kDa precursor that is then transported to the Golgi and lysosomes, where signal peptide cleavage and oligosaccharide trimming yield a 100 kDa mature homodimeric enzyme (with a mature monomeric molecular weight of 46 kDa) [2–4]. The loss of α-Gal A activity results in the progressive systemic accumulation of its substrate globotriaosylceramide (Gb3) and its deacylated derivative, globotriaosylsphingosine (Lyso-Gb3) [5, 6]. There are two main clinical subtypes of FD. The clinical manifestations of classic FD mainly include hypohidrosis, acroparesthesia, nonspecific gastrointestinal symptoms, angiokeratoma, and characteristic corneal dystrophy, which usually appear in childhood or adolescence. Classic FD typically affects males and results in essentially no residual α-Gal A activity. The natural history of the disease positively correlates with the progressive deposition of substrates. The accumulation of substrates can lead to cardiac, renal, and/or cerebrovascular diseases, which ultimately lead to a reduced lifespan (by approximately 10–20 years) and premature death [7, 8]. Individuals with late-onset FD do not develop early clinical features of classic FD due to low levels of mutated residual α-Gal A activity; however, complications are observed in the fourth to sixth decades and include cerebral vascular disease, as well as heart and kidney failure [9–11].

A standard treatment for patients with FD is enzyme replacement therapy (ERT), which involves regular intravenous infusions of human recombinant α-Gal A enzyme [12–14]. Through endocytosis mediated by mannose-6-phosphate receptors (M6P), the recombinant enzyme is absorbed by secondary tissues [15]. ERT is effective if rhα-Gal A is administered early in the disease process [16–18]. However, the relatively short half-life of rhα-Gal A means that infusions are required every 2 weeks, imposing a lifetime burden on the patient [13]. Additionally, there is also a significant percentage of FD patients who develop antibodies against rh-Gal A, which may reduce the effectiveness of treatment if they neutralize the enzyme [19, 20]. Migalastat, an oral pharmacologic chaperone, is also approved in multiple countries for the treatment of FD [21], however, it is only used for patients with amenable *GLA* mutations [22–25]. Therefore, the development of an effective, safe, and durable treatment is urgently required.

In the current field of gene therapy, recombinant adeno-associated virus (rAAV) vectors have shown great promise in both preclinical and clinical trials by effectively delivering therapeutic genes of interest to the liver and have shown a positive effect in treating hemophilia. Indeed, genes delivered by rAAV vectors persistently expressed factor IX in hemophilia B patients for up to 7 years [26]. A number of ongoing studies are evaluating the efficacy and safety of liver-targeted AAV gene therapy for various LSDs. AAV8-mediated liver delivery of secretable acid α-glucosidase was used for treating pathological glycogen accumulation in multiple tissues in Pompe disease [27]. It has been demonstrated that AAV8 provides a broader transduction area in the MPS IIIB mouse brain compared with AAV5, 9, or rh10, and that the treatment effect is better [28]. Liver-specific expression of G6Pase mediated by AAV2/8 vectors effectively reverses type Ia hypoglycemia in canine and murine glycogen storage diseases [29]. Previous preclinical evaluations of gene therapy approaches in FD mice (*Gla* KO) have shown that AAV2- and AAV1-mediated gene therapy results in suboptimal substrate clearance in key target tissues of FD mice, particularly in the kidney [30–32]. Although later use of AAV8 vector–mediated gene therapy significantly improved transgene expression in FD mice, the normalization of kidney Gb3 was only observed when FD mice were treated at a young age (1 month old) and before the onset of significant disease pathology [33]. The scAAV9-PGK-GLA vector has also been used to treat newborn FD mice through systemic injection [25]. Although this treatment method can pass the blood–brain barrier, it still requires further investigation before clinical application.

Herein, we describe a series of preclinical animal studies using a liver-targeted rAAV2/8 vector encoding human *GLA* cDNA driven by liver-specific promoters and enhancers. For the purpose of our study, *Cyegene* created the *Gla* KO mouse model (C57BL/6N) by CRISPR/Cas-mediated genome editing using a similar strategy by in report by Higuchi et al. [34]. In *Gla* KO mice, Gb3 accumulation was sharply elevated in the blood and various organs as a result of the *GLA* null mutation [35]. AAV2/8-hGLA gene therapy was evaluated in this mouse model of FD. A three-month detailed pharmacology and toxicology study was performed by injecting the AAV2/8-hGLA vector into the tail vein of FD mice at different doses. The results demonstrated that AAV2/8-hGLA efficiently infected the liver, leading to markedly increased plasma and tissue α-Gal A activities and essentially normalized Gb3 and Lyso-Gb3. Furthermore, the AAV2/8-hGLA vector treatment has not been associated with any obvious adverse effects in mice. The studies presented here demonstrate the feasibility of liver-directed gene therapy with AAV2/8-hGLA for FD in a preclinical animal model.

## Results

### Different doses of AAV2/8-hGLA lead to the continuous expression of α-Gal A with glycosylation and enzymatic activity in the livers of FD mice

**Fig. 1:**
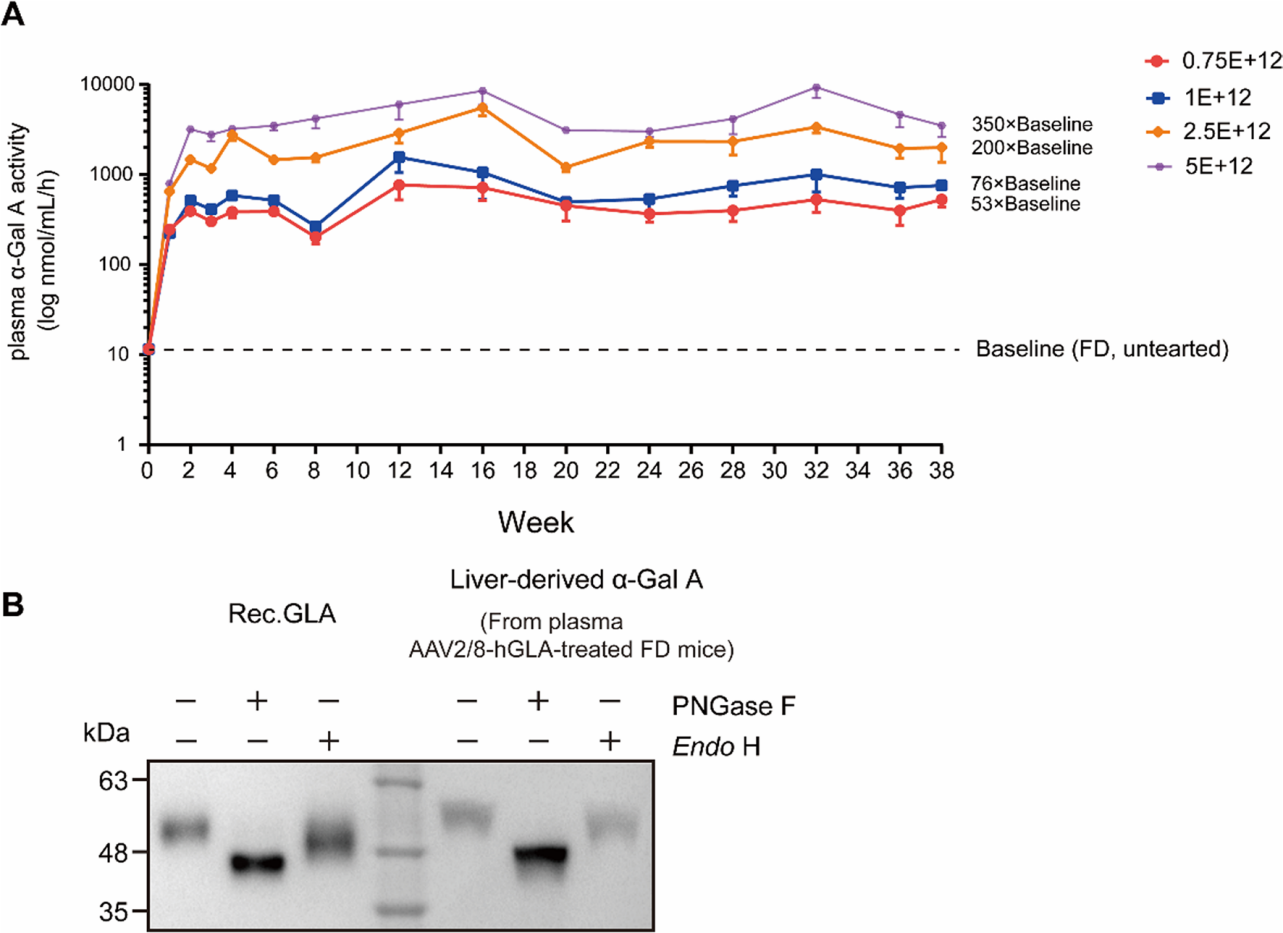
AAV2/8-hGLA produces continuous supraphysiological levels of glycosylation-modified α-Gal A in the plasma of FD mice. **A** α-Gal A activity was determined in the plasma of FD mice treated with different doses of AAV2/8-hGLA. Data are presented as the mean ± SEM (*n* = 2–12). Plasma was collected through tail vein bleeding at the indicated times, and the enzyme activity was measured using an α-Galactosidase (α-GAL) Activity Assay Kit. The dotted line indicates plasma α-Gal A at steady state levels in the untreated FD mice. **B** α-Gal A protein glycosylation patterns of plasma were determined in the plasma of 2.5E + 12 vg/kg AAV2/8-hGLA–treated FD mice; representative Western blot analyses are shown. Rec.GLA (an α-Gal A expressed in CHO mammalian cells) was used as a positive control for glycosylation analysis. PNGase F = peptide:N-glycosidase F; *Endo* H = endoglycosidase H

To evaluate the potential of gene therapy for FD, we generated an AAV2/8-hGLA containing a codon-optimized human *GLA* cDNA and liver-specific promoter and enhancer elements by a small-scale HEK293 production method, and assessed its pharmacodynamic activity in male FD mice. Compared with the FD group, α-Gal A activity was significantly increased in a dose-dependent manner and exhibited continued stable enzyme activity after reaching the highest enzyme activity in the second week of administration during the study period (Fig. 1A), indicating that the liver-expressed α-Gal A was active and continuously secreted into the bloodstream. At 38 weeks post-treatment, the plasma activity was 529.86 ± 91.90 nmol/h/mL in the lowest-dose group (0.75E + 12 vg/kg), which was significantly higher compared with FD mice levels. In contrast, the mean plasma activity in the highest-dose group (5E + 12 vg/kg) was 3515.00 ± 880.46 nmol/h/mL, corresponding to an increase of more than 350-fold compared with the mean FD mice levels.

The mature protein is comprised of two subunits of 398 amino acids (approximately 51 KDa), each of which contains three N-linked glycosylation sites. To assess the glycosylation status of plasma α-Gal A expressed by the AAV2/8-hGLA, plasma samples from 2.5E + 12 vg/kg AAV2/8-hGLA–treated FD mice were digested with PNGase F or *Endo* H and assessed via Western blot. As shown in Fig. 1B, AAV2/8-hGLA–expressed α-Gal A displayed deglycosylation patterns similar to those of Rec.GLA produced in CHO cells. These findings indicated appropriate glycosylation of AAV2/8-hGLA–expressed α-Gal A. Notably, AAV2/8-hGLA expresses a large number of glycosylation-modified α-Gal A precursors in the liver, which were secreted into plasma. The precursor α-Gal A has glycosylation modification, and it can be absorbed by various tissues, used to target lysosomes, and processed/modified into the 46 KDa lysosomal α-Gal A.

The above results showed that tail vein injection of AAV2/8-hGLA effectively transduced FD mouse livers, resulting in sustained long-term AAV2/8-mediated expression of glycosylation-modified α-Gal A in the plasma and effective expression of α-Gal A at a lower dose of AAV2/8-hGLA.

## Dose-dependent increases of vector DNA copy number and GLA mRNA levels in the livers of FD mice

**Fig. 2:**
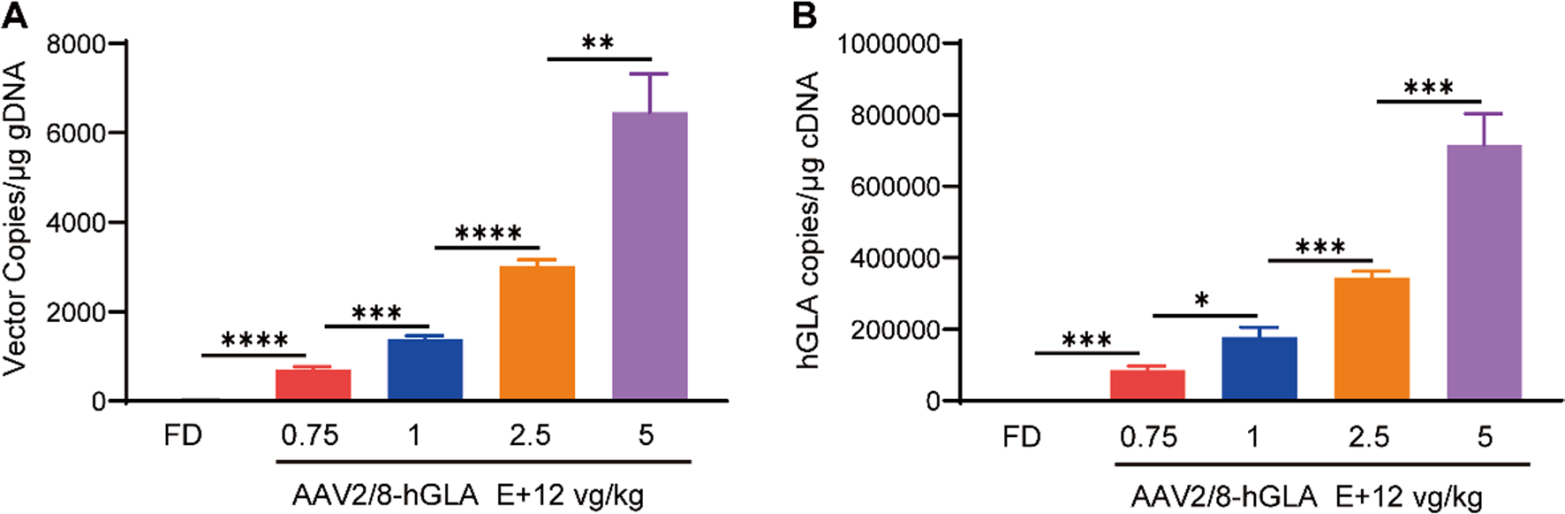
AAV2/8-hGLA copy numbers and the copy number of *GLA* mRNA in the livers of AAV2/8-hGLA–treated FD mice. FD mice were intravenously administered formulation buffer or the AAV2/8-hGLA at doses of 0.75E + 12, 1E + 12, 2.5E + 12, and 5E + 12 vg/kg, and tissues were collected at 12 weeks post-injection. **A** AAV vector genome (vg) copy numbers and **B** the copy number of *GLA* mRNA were determined in the liver. Data are presented as the mean ± SEM (*n* = 4, 5, 5, 6, and 4 for the FD, 0.75E + 12, 1E + 12, 2.5E + 12, and 5E + 12 vg/kg groups, respectively). *P < 0.05, **P < 0.01, ***P < 0.001, ****P < 0.0001. Unpaired *t-test* was used to compare two groups of data. gDNA = genomic DNA

We sought to design a high-efficiency expression vector to effectively treat FD at a low dose. Therefore, FD mice were intravenously administered formulation buffer or the AAV2/8-hGLA at doses of 0.75E + 12, 1E + 12, 2.5E + 12, and 5E + 12 vg/kg, and tissues were collected at 12 weeks post-injection. We next verified the infection efficiency of the vector and the level of *GLA* mRNA transcription. The qPCR results indicated dose-dependent increases in vector DNA copy numbers (Fig. 2A) and the copy number of *GLA* mRNA (Fig. 2B) in the liver after a 12-week administration of AAV2/8-hGLA at different doses. Compared with the FD group, vector DNA copy numbers (****P < 0.0001) and the copy number of *GLA* mRNA (***P < 0.001) in FD mice treated with 0.75E + 12 vg/kg AAV2/8-hGLA were significantly increased (Fig. 2). The results of vector DNA copy number and *GLA* mRNA copy numbers were consistent with the previous FD mouse plasma enzyme activity results, further confirming the effective therapeutic effect of AAV2/8-hGLA. The above results indicate that low-dose AAV2/8-hGLA (0.75E + 12 vg/kg) can effectively infect the liver, leading to the effective expression of α-Gal A.

## Expression of α-Gal A in the livers of AAV2/8-hGLA–treated FD mice

**Fig. 3:**
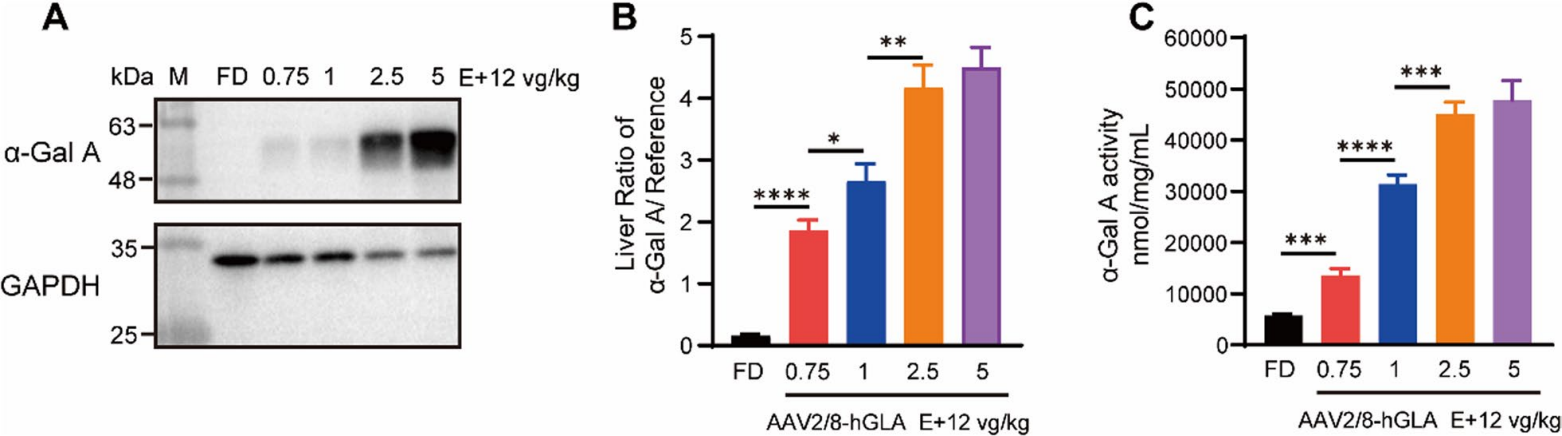
Expression of α-Gal A in the livers of AAV2/8-hGLA–treated FD mice. FD mice were intravenously administered formulation buffer or the AAV2/8-hGLA at doses of 0.75E + 12, 1E + 12, 2.5E + 12, and 5E + 12 vg/kg, and tissues were collected at 12 weeks post-injection. **A** α-Gal A protein content in livers of FD mice treated with different doses of AAV2/8-hGLA was determined 12 weeks after administration; representative Western blot analyses are shown, and **B** relative gray value analyses were performed. **C** α-Gal A activity was determined in livers of AAV2/8-hGLA-treated FD mice. α-Gal A enzyme activity was measured using the α-Galactosidase (α-GAL) Activity Assay. Data are presented as the mean ± SEM (*n* = 6, 6, 6, 6, 4, for the FD, 0.75E + 12, 1E + 12, 2.5E + 12, and 5E + 12 vg/kg groups, respectively). *P < 0.05, **P < 0.01, ***P < 0.001, ****P < 0.0001. Unpaired *t-*tests was used to compare two groups of data

Next, to verify the expression of α-Gal A in the liver of FD mice treated with different doses of AAV2/8-hGLA, we examined α-Gal A levels in the livers through Western blot and enzyme activity. The Western blot results showed that compared with the FD group, the expression of α-Gal A in the liver of FD mice in the 0.75E + 12 vg/kg treatment group was significantly increased, while the expression of α-Gal A in different-dose treatment groups was dose-dependently increased (Fig. 3A and B). The results of enzyme activity detection (Fig. 3C) were consistent with those of Western blot. Combined with the above results, these findings demonstrate that lower doses of AAV2/8-hGLA can effectively infect the liver of FD mice to express α-Gal A and secrete it into the plasma.

## α-Gal A enzyme secreted from AAV2/8-hGLA–treated FD mouse liver into the bloodstream is taken up by distal secondary tissues

**Fig. 4:**
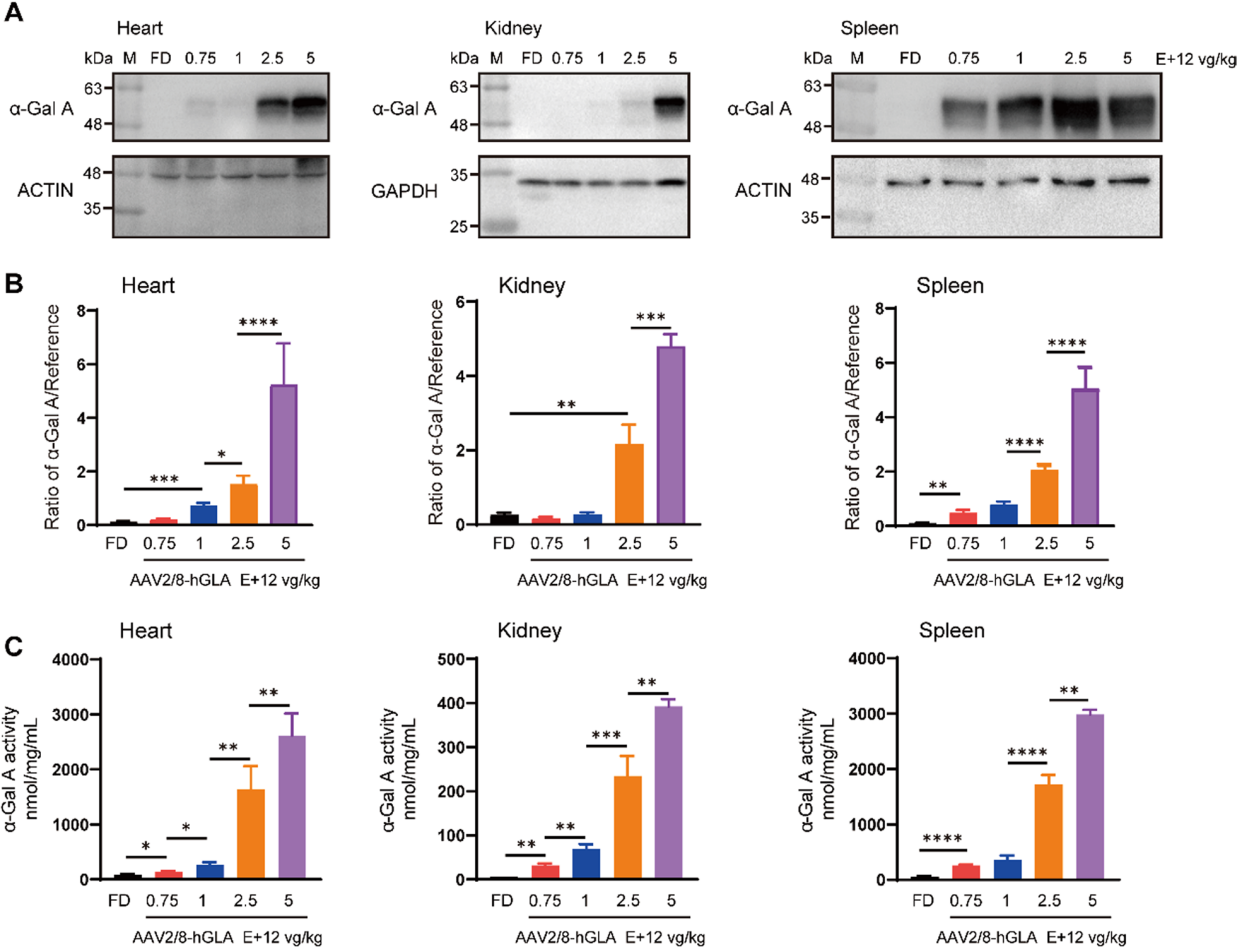
Uptake of α-Gal A in various tissues of AAV2/8-hGLA–treated FD mice. FD mice were intravenously administered formulation buffer or the AAV2/8-hGLA at doses of 0.75E + 12, 1E + 12, 2.5E + 12, and 5E + 12 vg/kg, and tissues were collected at 12 weeks post-injection; representative Western blot analyses are shown. **A** α-Gal A protein content in tissues of FD mice treated with different doses AAV2/8-hGLA determined 12 weeks after administration, and **B** relative gray value analyses were performed. **C** α-Gal A activity was determined in different tissues of the treated FD mice. α-Gal A enzyme activity was measured using the α-Galactosidase (α-GAL) Activity Assay Kit. Data are presented as the mean ± SEM (*n* = 4–6). *P < 0.05, **P < 0.01, ***P < 0.001, ****P < 0.0001. Unpaired *t*-tests were used to compare two groups of data

Based on the abovementioned results, we further examined whether the active α-Gal A expressed in the liver could be effectively taken up by various tissues. Cardiomyocytes, glomeruli, and renal tubules are major stores of Gb3 and Lyso-Gb3. Importantly, accumulation of Gb3 in the glomerulus causes kidney disease, leading to glomerulosclerosis and eventually kidney failure. The efficient uptake of α-Gal A by these cells is the key of the AAV2/8-hGLA therapeutic effect. We verified α-Gal A content in various tissues of FD mice treated with different doses of AAV2/8-hGLA using Western blot and enzyme activity detection. The results of Western blot and enzyme activity detection showed that the uptake of α-Gal A in each tissue of FD mice increased with the increase in the administration dose (Fig. 4). Compared with the FD group, the α-Gal A activity in the heart, kidney, and spleen was significantly increased in the 0.75E + 12 vg/kg dose group (Fig. 4C). Compared with the 2.5E + 12 vg/kg and 5E + 12 vg/kg dose groups, the uptake of α-Gal A in the heart, the spleen and the kidney, but not in the liver, was significantly different. These results indicate that the active α-Gal A expressed in the livers of FD mice following AAV2/8-hGLA treatment can be effectively absorbed by various tissues, which lays the foundation for the efficient removal of the storage substrates Gb3 and Lyso-Gb3.

## α-Gal A enzyme secreted from AAV2/8-hGLA effectively clears the accumulated substrates Gb3 and Lyso-Gb3

**Fig. 5:**
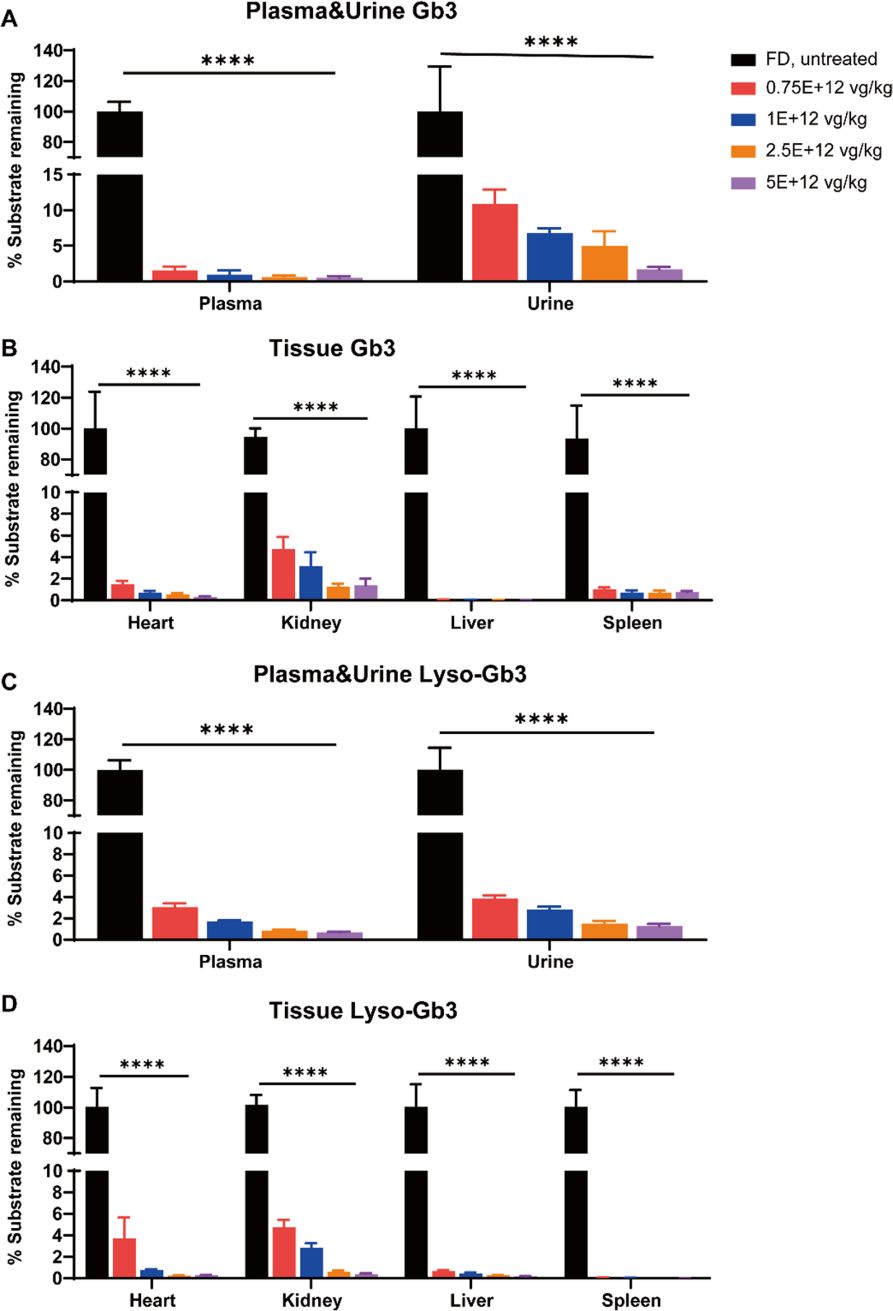
Levels of Gb3 and Lyso-Gb3 in plasma, urine, and organs of FD mice treated with different doses of AAV2/8-hGLA. FD mice were intravenously administered formulation buffer or AAV2/8-hGLA at doses of 0.75E + 12, 1E + 12, 2.5E + 12, and 5E + 12 vg/kg, and tissues were collected at 12 weeks post-injection. Efficacy of different doses of AAV2/8-hGLA administration in clearing storage substrates Gb3 and Lyso-Gb3 in different tissues of FD mice. Data are presented as the mean ± SEM (*n* = 3–7). Data were analyzed using one-way ANOVA followed by Tukey’s multiple comparison tests. ****P < 0.0001, comparison of different doses of AAV2/8-hGLA treatment and FD group

Detection of the content of Gb3 and Lyso-Gb3 in plasma and urine is the current gold standard for clinical diagnosis of patients with FD, and the heart, kidney, liver, and spleen are key organs in FD pathology. Therefore, we measured the concentrations of Gb3 and Lyso-Gb3 in these tissues, plasma, and urine 12 weeks after treatment so as to determine whether α-Gal A secreted from the liver into the blood could effectively clear the accumulated substrates (Fig. 5). The LC–MS/MS results showed that compared with the FD group, the plasma, urine, and tissue substrate concentrations of FD mice in the 0.75E + 12 vg/kg AAV2/8-hGLA treatment group were significantly reduced, and in all AAV2/8-hGLA treatment groups tested, Gb3 and Lyso-Gb3 levels were ≤ 10%. Moreover, the substrate concentrations in these tissue sites of FD mice were inversely proportional to the increasing dose of vehicle.

For further verification that Gb3 could be cleared in which type of cells, immunohistochemical analysis of α-Gal A on the heart and renal was performed. The result showed that the AAV2/8-hGLA treatment resulted in abundant α-Gal A presence in cardiomyocytes, renal tubular cells, and podocytes of FD mice, as expected (Additional file 1: Fig. S1).

## Discussion

In recent years, the safety and efficacy of AAV as a vector for gene therapy have been validated by clinical trials of AAV-mediated liver-targeted therapy for hemophilia A and B [36–38]; consequently, in vivo clinical applications of AAV-based vectors for liver-targeted gene therapy are rapidly increasing. Liver-targeted gene therapies for FD, namely FLT190 [39] and ST-920 [40], are both in phase 1/2 clinical research, and both have achieved good safety and efficacy. Therefore, we attempted to design a highly efficient AAV2/8-hGLA to establish a proof-of-concept for the clinical development of liver-targeted gene therapy for FD so as to improve the therapeutic effect and reduce the dose of AAV vector, thereby reducing the cost of gene therapy.

We validated the therapeutic effect of liver-targeted AAV2/8-hGLA in FD mice, which was produced using the HEK293 scientific research-scale manufacturing process. The results showed that α-Gal A activity significantly increased in FD mouse plasma and tissue. Indeed, the α-Gal A activity in the FD mouse livers was significantly higher than that in the FD group under the treatment of 0.75E + 12 vg/kg AAV2/8-hGLA (*p* = 0.0001), which resulted in a large amount of α-Gal A being secreted into plasma. The α-Gal A activity in the 0.75E + 12 vg/kg AAV2/8-hGLA–treated FD mouse plasma was approximately 53-fold higher than that of the FD group. The above results showed that AAV2/8-hGLA treatment results in high expression of α-Gal A in the livers of FD mice. The entire experiment was performed in the absence of immunosuppression, and following a single tail vein injection, the plasma enzyme activity in FD mice significantly increased in a dose-dependent manner, reaching a plateau in the second week and then maintaining sustained and stable plasma enzyme activity for 38 weeks.

After 12 weeks of AAV2/8-hGLA treatment, we found no significant difference in the plasma levels of aspartate aminotransferase (AST) and alanine aminotransferase (ALT) in FD mice treated with different doses of AAV2/8-hGLA (data not shown). The highest dose of AAV2/8-hGLA was 5E + 12 vg/kg, but no adverse reaction was observed in this group, confirming the safety of AAV2/8-hGLA. Compared with the FD group, at the dose of 0.75E + 12 vg/kg, the content and enzyme activity of α-Gal A in the tissues of FD mice were significantly increased in a dose-dependent manner. Reports in the literature show that as long as 5%–10% of α-Gal A enzyme activity remains, Gb3 and Lyso-Gb3 storage substrates can be effectively removed [41], indicating that the α-Gal A expressed in the AAV2/8-hGLA–treated FD mouse liver is effectively secreted into the plasma and taken up in FD mouse tissues, especially by the heart and kidney. Studies conducted earlier have shown that female FD mice exhibit reduced efficiency of transduction and transgene expression of AAV in comparison to males [25, 32, 39]. The effect may be caused by sex hormones, however, other studies pointed out that, unlike mice, androgens did not affect the transgene expression of AAV in nonhuman primates (NHPs) [42]. Thus, it is still necessary to determine if there exists a disparity in AAV2/8-hGLA transgenic expression between male and female FD mice. Although *Gla* KO mice have metabolomics profiles of FD, they do not exhibit the abnormal behavioral phenotype during our entire experiment cycle (from weeks 12 to 24). Despite this, a variety of preclinical pharmacokinetic and pharmacodynamic studies have been conducted on such models to evaluate enzyme replacement, substrate reduction, and gene therapy strategies for FD [43–46].

FD occurs as a result of the increased accumulation of Gb3 and Lyso-Gb3 in lysosomes, which leads to various complications [1]. Indeed, the storage content of substrates in patients is the current gold standard for the diagnosis and treatment of FD [47]. Our study showed that the content of Gb3 and Lyso-Gb3 in the plasma, urine, and tissues (heart, kidney, liver, and spleen) of FD mice treated with AAV2/8-hGLA was significantly reduced and tended to normalize compared with the FD group. Previous studies have shown that cardiac and renal failure are the most common and life-threatening complications in the end stage of most patients with FD disease; the main reason for the renal complications observed in FD is the poor clearance of Gb3, while the reason for the cardiac complications is the accumulation of Lyso-Gb3 [48]. Therefore, it is important to pay special attention to the clearance of renal Gb3 and cardiac Lyso-Gb3 when developing new therapies. Previous studies on FD have shown that poor clearance of substrate storage in the kidney is an important factor restricting the therapeutic effect on FD. However, our study showed that in FD mice treated with 0.75E + 12 vg/kg AAV2/8-hGLA, the residual Gb3 storage substrate in urine and kidney was < 10%, and the residual Lyso-Gb3 in heart was < 5%, indicating that AAV2/8-hGLA at a low dose had a good therapeutic effect in FD. And not only that the AAV2/8-hGLA treatment resulted in abundant α-Gal A presence in cardiomyocytes, renal tubular cells, and podocytes of FD mice. This could be taken as an indication that the Gb3 and Lyso-Gb3 could be efficiently cleared in these cells.

Notably, previous preclinical evaluations of AAV1- and AAV2-mediated gene therapy approaches in FD mice have shown suboptimal substrate clearance in key target tissues, particularly in the kidney [30–32]. Although the use of AAV8 vectors significantly improved transgene expression, normalization of renal Gb3 was only achieved when FD mice were treated at a young age (1 month old), before the onset of significant disease pathology [33]. The reason for this result may be related to the immune response, and the systemic expression of α-galactosidase using a constitutive promoter induces a strong immune response at low levels. The capsid, its genome, and transgene products are the main potentially immunogenic components of AAV vectors [49]. Other host-dependent and vector-dependent factors can modulate overall vector immunogenicity [49]. However, previous studies have shown that in the context of liver-directed gene transfer, transgene immunogenicity does not appear to be a problem compared with other tissues. Since the initial observation that mice expressing human factor IX in the liver are immune to transgenic products, several studies using AAV vectors in small and large animal models of genetic disease have shown that expression of the antigen in hepatocytes can promote strong antigen-specific immune tolerance [50–52]. Despite the success of AAV2/8-hGLA in clearing glycosphingolipids from peripheral organs, the enzyme generated is not expected to cross the blood–brain barrier (BBB). In the ensuing plan, we will develop a modified α-Gal A protein with a brain-targeting peptide in the future to achieve BBB transport and alleviate brain pathology and behavioral deficits [53].

Based on the results of previous studies, the infection efficiency and expression elements of the viral vector are the main factors affecting the therapeutic effect of gene therapy. In line with this, we aimed at improving the design of the transgene expression cassette to further enhance the AAV2/8-hGLA–mediated α-Gal A expression in plasma and tissues. As the *GLA* cDNA sequence used in AAV2/8-hGLA was optimized, further efforts have focused on engineering the AAV serotype to minimize the immune response elicited by the AAV vector. In vitro studies have demonstrated that AAV plasmid can effectively transfect liver cell and expression α-Gal A. In vivo studies have demonstrated that AAV2/8-hGLA can effectively transfect FD mouse livers, resulting in the expression and secretion of biologically active α-Gal A with high efficiency. Maximizing the efficacy of therapeutic AAV vectors is critical for clinical applications. Indeed, previous studies have shown that transduction of hepatocytes is more challenging in large animals, such as non-human primates (NHPs), compared with mice, with a 50- to 100-fold reduction in transduction efficiency [42, 54]. Furthermore, a more potent vector would allow to use lower vector doses, potentially allowing the vector to circumvent or minimize the anti-AAV capsid immune response associated with the administration of higher vector doses [54]. Notably, previous studies in transgenic mice overexpressing α-Gal A have demonstrated the safety of elevated systemic α-Gal A activity levels of up to 155- and 44-fold in the plasma and liver, respectively [55].

AAV2/8-hGLA (a safe and effective gene therapy for liver-specific expression) has several advantages over ERT (the current standard of care for FD): ① Liver-mediated AAV gene therapy has the potential to induce immune tolerance to the therapeutic target protein, while the formation of neutralizing antibodies against recombinases remains a limiting factor for the therapeutic effect of ERT. ② Gene therapy may become a “one-time treatment, life-long cure” treatment method for patients with FD, while ERT requires life-long treatment of patients, infusions of 2–4 h every 2 weeks, resulting in physical and psychological burdens for patients [19, 20, 56]. Furthermore, in mouse models of hemophilia B and Pompe disease, AAV-mediated gene therapy using preexisting antibodies against the respective therapeutic proteins resulted in sustained reduction or elimination of antibodies [43, 57, 58]. If true in humans, this would be a significant advantage, as it would allow the use of AAV gene therapy to treat patients with FD with preexisting α-Gal A antibodies (generated by ERT). The successful application of the AAV2/8 vector in FD mice has laid a theoretical foundation for the use of AAV2/8-mediated liver-specific expression gene therapy to treat LSDs. We will create a LSD gene therapy platform that we expect to reduce the dose and treatment cost of AAV vectors, thereby bringing hope for the realization of a one-time treatment for patients with LSDs.

## Conclusions

In conclusion, this preclinical study demonstrated the feasibility of expressing durable and therapeutic levels of α-Gal A using a research-scale manufactured AAV2/8-hGLA. The highly optimized AAV2/8-hGLA yielded the highest plasma and tissue transgene expression and demonstrated a favorable safety profile in FD mice. Taken together, these findings lay the groundwork for a phase I/II clinical trial to evaluate the safety and efficacy of AAV2/8-hGLA, a liver-targeted AAV2/8-mediated gene therapy approach, in patients with FD.

## Materials and methods

### AAV2/8 vector production

The AAV2/8-hGLA vector is a transgenic expression cassette vector containing a liver-specific promoter and enhancer, codon-optimized full-length human *GLA* cDNA, and a transgenic expression cassette including the native *GLA* signal peptide (amino acids 1–31). The AAV2/8-hGLA was produced using a manufacturing system: triple transfection system in HEK cells. The AAV2/8-hGLA was provided by Caygen Biosciences Inc. (Guangzhou, China).

## Preclinical studies in the FD mice

C57BL/6 male mice aged 8 weeks were purchased from the Model Animal Research Center of Nanjing University. Female heterozygous FD mice (*Gla*-knockout mice, G*la*^+/−^) were purchased from Cyagen (C57BL/6N-*Gla*^em1Cya^, KOCMP-11605-*Gla*, Suzhou, China); the male wild-type mice were mated with the female heterozygous FD mice to obtain male hemizygous FD mice to evaluate the therapeutic effect of AAV2/8-hGLA. All mice were housed at 22℃ with a 12-h light–dark cycle under specific pathogen-free conditions. Food and water were provided ad libitum. All animal experiments were conducted with the approval from the Institutional Animal Care and Use Committee and the animal housing facility of the South China University of Technology (Guangzhou, PR China, Ethics permit number: 2021065).

Five different dose AAV2/8-hGLA-treated groups were designed as follows: FD (0.0E + 12 vg/kg), 0.75E + 12 vg/kg, 1E + 12 vg/kg, 2.5E + 12 vg/kg, and 5E + 12 vg/kg. The FD group was injected with preparation buffer through the tail vein, while the AAV treatment group was injected with a different dose of AAV2/8-hGLA through the tail vein. The total injection volume for each mouse was 200 μL. To assess the kinetics and durability of transgene expression, after the first tail vein administration, the plasma enzyme activity of the mice was measured by weekly tail blood samples for 4 weeks, every 2 weeks for 4–8 weeks, and every 4 weeks after 8 weeks. Blood samples were collected in EDTA anticoagulant tubes, centrifuged at 4 °C and 2,700 × g for 10 min to separate plasma, and stored at− 80 °C for later use. After caudal vein administration, they were observed for 12 weeks, the mice were sacrificed at the indicated times by perfusion with phosphate-buffered saline (PBS) via the left ventricle under tribromoethanol anesthesia. Next, the organs (heart, liver, kidney, and spleen) were split into two parts and divided into two tubes: a part in which the tissues were snap-frozen in liquid nitrogen and stored at − 80 °C until biochemical assays and related molecular experiment, and another part in which the tissues were fixed using paraformaldehyde (PFA; 4% v/v).

## α-Gal A enzymatic activity assay

α-Gal A activity was determined using a spectrophotometer. The α-Galactosidase (α-GAL) Activity Assay Kit for enzyme activity analysis was obtained from Solarbio (BC2570, Guangzhou, China). The collected plasma was diluted 40 × , after which enzyme assays were performed according to the manufacturer’s instructions.

Briefly, tissues (~ 30 mg tissue per sample) were embedded in deionized water (300 μL) and homogenized with freezing grinding apparatus (JXFSTRPR-CL). The preparations were centrifuged at 10,000 × *g* to collect cytosolic fractions. The heart and kidney cytosolic fractions were diluted 10 × to assay the enzyme activities, while the liver and spleen cytosolic fractions were diluted 20 × . The enzyme activity assay was prepared in accordance with the manufacturer’s instructions. The total protein concentration was measured with the Pierce™ BCA Protein Assay Kit (Thermo Fisher Scientific, Waltham, MA, USA) following the manufacturer’s protocol. The specific activity was expressed as nanomoles of hydrolyzed substrate per hour and milligrams of total protein.

## Plasma α-Gal A glycosylation analysis

Aliquots of the plasma α-Gal A were digested with peptide:N-glycosidase F (PNGase F, #P0704S; New England Biolabs, Ipswich, MA, USA) or endoglycosidase H (*Endo* H, #P0702S; New England Biolabs, Ipswich, MA, USA), following the manufacturer’s protocol. Then, a 1:6 enzymatic hydrolysis solution and loading buffer were mixed, denatured at 95 °C for 10 min, and stored at − 20 °C; the glycosylation patterns of plasma α-Gal A were verified via Western blot. Recombinant Human alpha-Galactosidase A/GLA Protein, CF (Rec. GLA, Catalog No. 6146-GH-020) was obtained from R&D Systems (Minneapolis, MN, USA), and Rec.GLA (an α-Gal A expressed in CHO mammalian cells) was used as a positive control for glycosylation analysis.

## Genomic DNA purification and pector genome quantitation

Genomic DNA was isolated from the mouse livers (~ 15 mg tissue per sample) using the FastPure Cell/Tissue DNA Isolation Mini Kit (DC102, Vazyme, China), in accordance with the manufacturer’s instructions. The concentration of DNA was determined using ScanDrop^2^ (AnalytikJena, Germany), and DNA samples at 50 ng/µL were used for qPCR analysis. Quantitative PCR was performed using ChamQ Universal SYBR qPCR Master Mix (Q711-02, Vazyme, China). The AAV2/8-hGLA genome copy number was determined using primers that bind specifically to promoter sequences (forward primer: 5’-CTCCAACATCCACTCGACCC-3’, reverse primer: 5’-GTACCACTTAGCTGGCCCTC-3’), and were detected by qTOWER3G IVD (AnalytikJena, Germany). PCR amplification was performed using the following cycling conditions: 2 min at 95 °C, followed by 40 cycles of 5 s at 95 °C and 20 s at 60 °C. Quantitation of the AAV2/8-hGLA genome was achieved using a dilution curve (1 × 10^7^ to 1 × 10^2^ copies per reaction) by preparing a pAAV-hGLA plasmid. Data were processed using qPCRsoft4.1 (AnalytikJena, Germany) software, and 96 well plates for qPCR analysis were purchased from NEST Biotechnology Co. Lab (402,712, Wuxi, China).

## Quantification of GLA mRNA in livers

Total RNA was purified from homogenized liver tissue using the E.Z.N.A.® Total RNA Kit I (R6834-01, Omega, China), and cDNA was synthesized using 5 × qRT Super Mix II following the manufacturer’s protocol. qPCR was performed using ChamQ Universal SYBR qPCR Master Mix (Q711-02, Vazyme, China) and a custom primer mix targeting the AAV2/8-hGLA *GLA* cDNA transgene (forward primer: 5’-AGGCTGGAAAGATGCTGGGTA-3’, reverse primer: 5’-TCGGATGCCATGTGG AAAGC-3’). Quantitation of the vector-specific hGLA mRNA copy number was achieved using a dilution curve (1 × 10^7^ to 1 × 10^2^ vg per reaction) with overexpression plasmid pAAV-hGLA. qPCR amplification was performed as outlined above.

## Expression and content of human α-Gal A in tissue lysates

The relative content of α-Gal A in tissues was determined by Western blot. Briefly, tissues (~ 30 mg tissue per sample) were homogenized using a freezing grinding apparatus (JXFSTRPR-CL, China) in RIPA lysis buffer (P0013B; Beyotime, China) containing protease and phosphatase inhibitor cocktail (P1046; Beyotime), and centrifuged for 20 min at 10,000 × g (4 °C) to collect cytosolic fractions. Protein concentration was determined with a BCA kit. The immunoprecipitated protein was then denatured by adding 6 × loading buffer and heated to 95 °C for 10 min. Proteins (~ 20 ug tissue per sample) were separated through SDS-PAGE and transferred to 0.45 μm polyvinylidene fluoride (PVDF) membranes (#ISEQ00010, Millipore, St. Louis, MO, USA). The membranes were blotted with a monoclonal anti-human-α-Gal A antibody (#ab168341; Abcam, Cambridge, UK) and a monoclonal β-actin (#A2228, Sigma-Aldrich, St. Louis, MO, USA) or GAPDH antibody (#G8795, Sigma-Aldrich). Following five washes, proteins were visualized using ECL reagent (WBKLS0500, Millipore) and images were captured using a Bio-Rad scanner (ChemiDoc XRS + system, Bio-Rad). The liver, heart, kidney, and spleen tissues of mice in each group were analyzed by WB. Each antigen band was normalized to its total protein lane using the total protein normalization tool (ImageJ) and following the manufacturer’s protocol. The different doses for each group were assigned as follows: FD (no AAV, n = 6), 0.75E + 12 vg/kg (n = 6), 1E + 12 vg/kg (n = 6), 2.5E + 12 vg/kg (n = 6), and 5E + 12 vg/kg (n = 4). A representative WB image for each organ was taken from a randomly selected animal in each dosing group.

## Gb3 and Lyso-Gb3 analyses in plasma, urine, and tissue samples

To measure Gb3 and Lyso-Gb3 scavenging efficiency, ceramide trihexoside (Gb3, 1067), lyso-ceramide trihexoside (Lyso-Gb3, 1520), N-heptadecanoyl-lactosylceramide (N-C17, 1538), and glucosylsphingosine (GLU, 1310) were purchased from Matreya (Pleasant Gap, PA) for liquid chromatography with tandem mass spectrometry (LC–MS/MS) analysis. Gb3 and Lyso-Gb3 were extracted from plasma, urine, and organs (heart, kidney, liver, and spleen) as follows. Briefly, plasma (50 μL), urine (100 μL), and tissue samples (5–15 mg) were mixed thoroughly or homogenized (tissue) in 600 μL methanol (containing internal standard: 40 ng N-C17&40 ng GLU). After addition of chloroform (300 μL) and ddH_2_O (100 μL), the samples were shaken and mixed thoroughly before centrifuging for 10 min at 18,630 g, after which the supernatant was transferred to another 10-mL glass tube. Next, the samples were dried in a nitrogen blower and resuspended in 200 μL methanol for LC–MS/MS analysis. Then, 5 μL of the processed sample was injected into a Thermo Scientific Ultimate 3000 liquid phase system and a liquid chromatography-triple quadrupole mass spectrometer (TSQ Endura, Thermo Fisher Scientific, Waltham, MA, USA) for LC–MS/MS analysis. The Gb3 of mixture was separated on a Thermo Accucore aQ C18 (150 × 2.1 mm, 2.6 μm; Thermo Fisher Scientific). The analytes were detected in positive ionization mode with the following transition: for Gb3, 1136.7 → 632.7. The Lyso-Gb3 of mixture was separated on a Hypersil Gold C18 column (50 × 2.1 mm, 1.9 μm; Thermo Fisher Scientific). The analytes were detected in positive ionization mode with the following transition: for Lyso-Gb3, 786.7 → 264.3. A Thermo Scientific Xcalibur was used for data acquisition and processing.

## Statistical analysis

Statistical analyses were performed using unpaired two-tailed Student’s *t*-tests or one-way ANOVA followed by Tukey’s multiple comparison tests in GraphPad Prism software version 8.4.

### Supplementary Information

**Additional file 1: Word S1** All data supporting the findings of this study are available within the paper and its Supplementary Information. IHC supplementary result and represent Wetern blot original picture are provided in additional file 1.

## Data Availability

All data generated or analyzed during this study are included in the article, further inquiries can be directed to the corresponding author. URLs is not applicable to this article as our study is preclinical animals and excludes clinical research and pertinent database analysis, etc.

## Abbreviations

FD: Fabry disease
α-Gal A: α-Galactosidase A
Gb3: Globotriaosylceramide
Lyso-Gb3: Globotriaosylsphingosine
ERT: Enzyme replacement therapy
AAV2/8: Adeno-associated viral 2/8
AAV2/8-hGLA: AAV2/8-hGLA vectors
LSDs: Lysosomal storage diseases
M6P: Mannose 6-phosphate
rAAV: Recombinant adeno-associated virus
PNGase F: Peptide:N-glycosidase F
*Endo* H: Endoglycosidase H
