# Supplementary material for: Systematic gene therapy derived from an investigative study of AAV2/8 vector gene therapy for Fabry disease
Source: Orphanet J Rare Dis. 2023 Sep 5;18:275. doi: 10.1186/s13023-023-02894-0 (PMC10481556; doi:10.1186/s13023-023-02894-0)
Supplement: Supplementary file 1 — Additional file 1: Word S1 All data supporting the findings of this study are available within the paper and its Supplementary Information. IHC supplementary result and represent Wetern blot original picture are provided in additional file 1. [file 13023_2023_2894_MOESM1_ESM.docx]

**Supplementary Information**

Materials and methods

**Immunohistochemistry(IHC)**

Tissues were fixed in formalin, embedded in paraffin, and cut into 5-μm-thick sections. In brief, after heat-induced epitope retrieval in EDTA buffer, the sections were treated with 3% hydrogen peroxide and 10% normal goat serum, before incubating with rabbit monoclonal antibody to human α-Gal A (Abcam). After incubation with HRP-labeled secondary antibody, signals were detected by DAB chromogen, and the sections were counterstained with hematoxylin. The signal specificity was verified with control staining, in which the primary antibody incubation was omitted. Compared with light and diffuse nonspecific staining in untreated FD group, the specific signal displayed a granular cytoplasmic pattern, consistent with the findings of a previous study.

Results

For further verification that Gb3 could be cleared in which type of cells, immunohistochemical analysis of α-Gal A on the heart and renal was performed. The result showed that the AAV2/8-hGLA treatment resulted in abundant α-Gal A presence in cardiomyocytes, renal tubular cells, and podocytes of FD mice, as expected (Fig. S1).


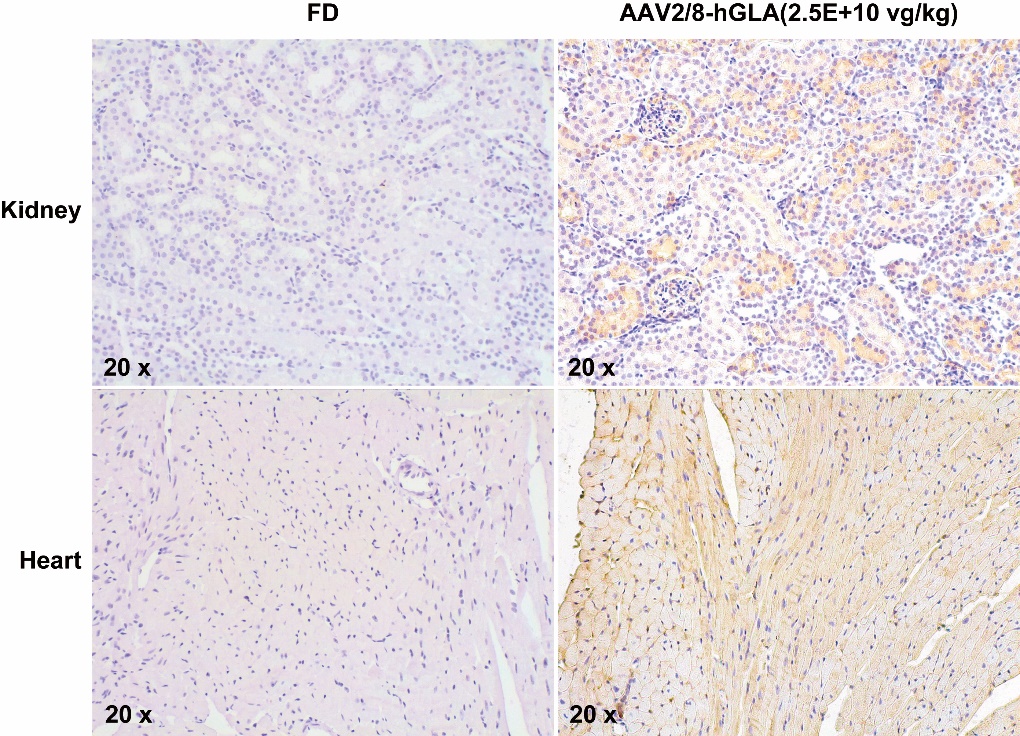


Supplemental Fig. S1 IHC staining was conducted on paraffin sections of 2.5E+12 vg/kg AAV2/8-hGLA treated FD mice various tissues using an anti-human α-Gal A antibody.

**WB original picture**

**A ①Fig3A-α-Gal A 50 KDa, Liver ②Fig3A-GAPDH 36 KDa, Liver**


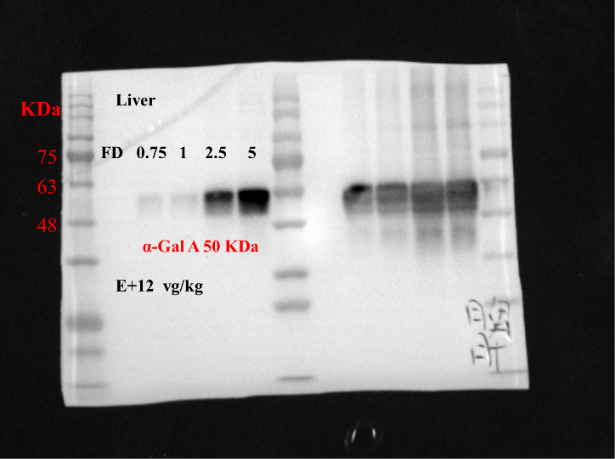

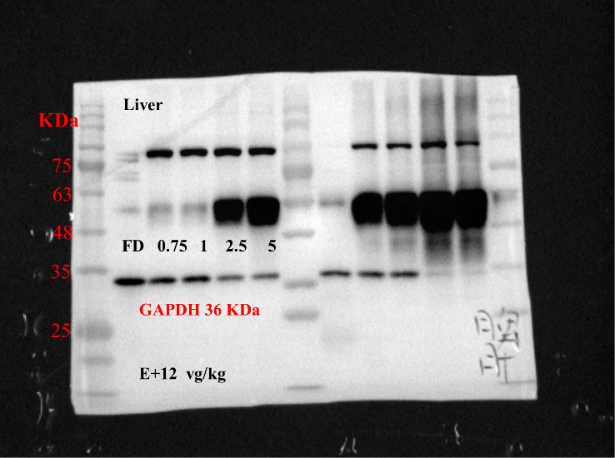


**B ③Fig4A-α-Gal A 50 KDa, Heart ④Fig4A-ACTIN 42 KDa, Heart**


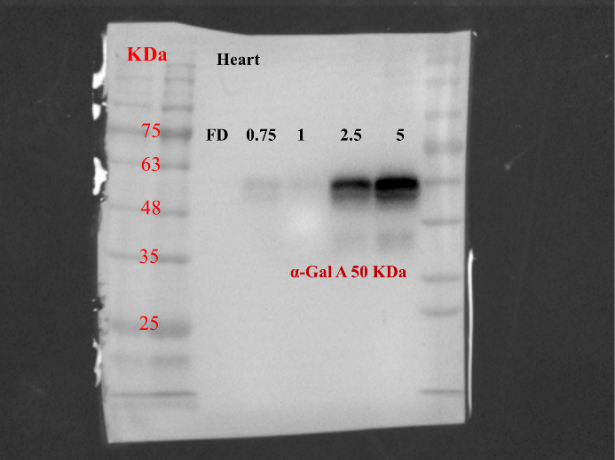

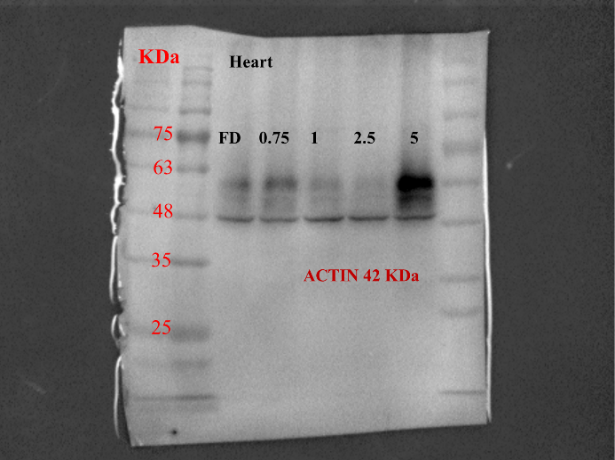


**C ⑤Fig4A-α-Gal A 50 KDa, Kidney ⑥Fig4A-GAPDH 36 KDa, Kidney**

**
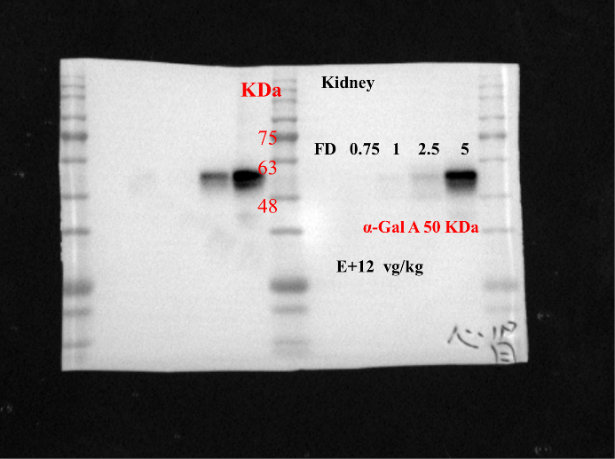
**
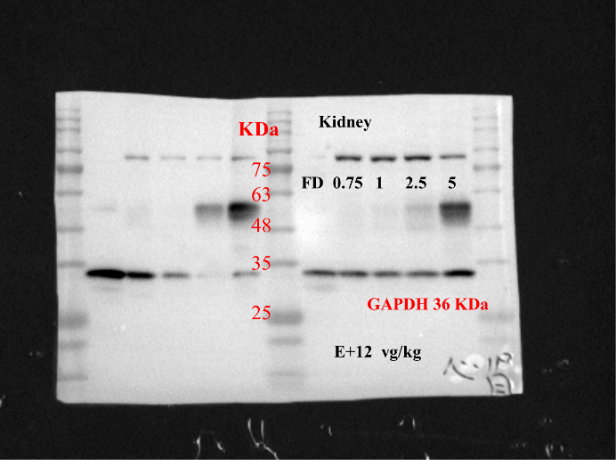


**D ⑦Fig4A-α-Gal A 50 KDa, Spleen ⑧Fig4A- ACTIN 42 KDa, Spleen**


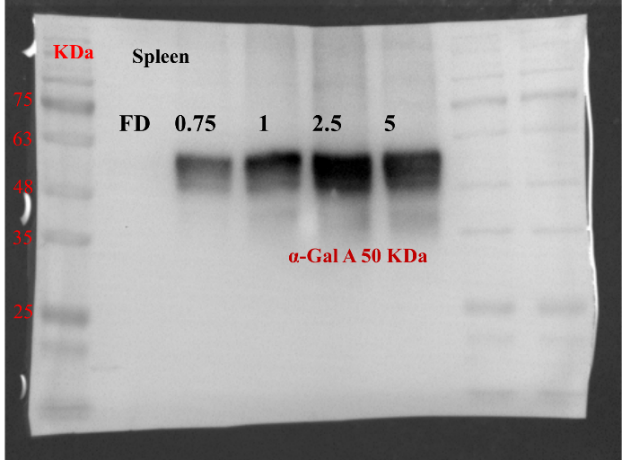

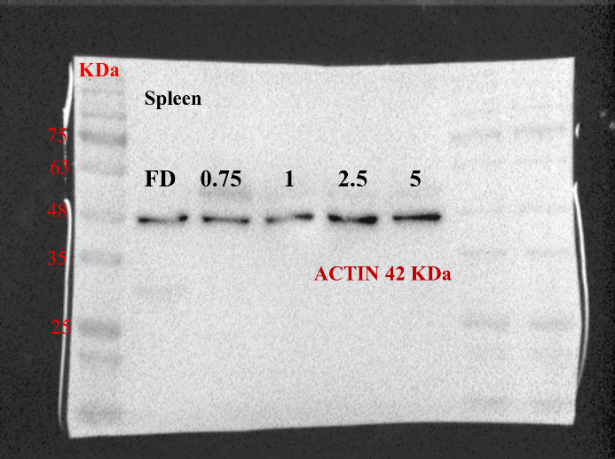


Supplemental Fig. 2. Western Blot analyses of α-Gal A in the tissues of AAV2/8-hGLA–treated FD mice. Represent western blot analysis of α-Gal A in the liver (A), in the heart (B), in the kidney (C) and in the spleen (D) of AAV2/8-hGLA–treated FD mice.


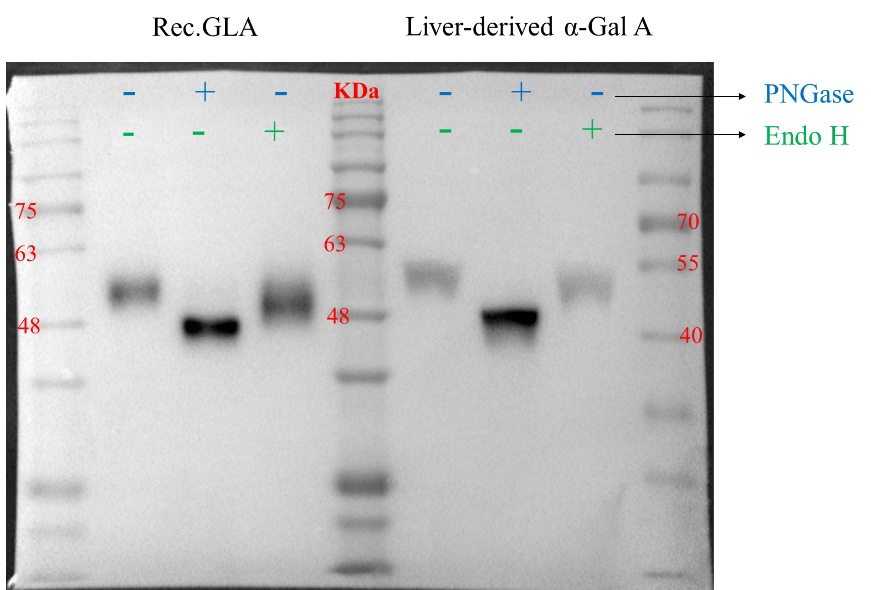


Supplemental Fig. 3. Western Blot analyses of α-Gal A glycosylation validation. α-Gal A protein glycosylation patterns of plasma were determined in the plasma of 2.5E + 12 vg/kg AAV2/8-hGLA–treated FD mice; representative Western blot analyses are shown. Rec.GLA (an α-Gal A expressed in CHO mammalian cells) was used as a positive control for glycosylation analysis. PNGase F = peptide:N-glycosidase F; *Endo* H = endoglycosidase H.
